# InterNet: Detection of Active Abdominal Arterial Bleeding Using Emergency Digital Subtraction Angiography Imaging With Two-Stage Deep Learning

**DOI:** 10.3389/fmed.2022.762091

**Published:** 2022-06-29

**Authors:** Xiangde Min, Zhaoyan Feng, Junfeng Gao, Shu Chen, Peipei Zhang, Tianyu Fu, Hong Shen, Nan Wang

**Affiliations:** ^1^Department of Radiology, Tongji Hospital, Tongji Medical College, Huazhong University of Science and Technology, Wuhan, China; ^2^College of Biomedical Engineering, South-Central of University for Nationalities, Wuhan, China; ^3^United Imaging Intelligence, Shanghai, China

**Keywords:** abdominal arterial bleeding, digital subtraction angiography, deep learning, automatic detection, two-stage model

## Abstract

**Objective:**

Active abdominal arterial bleeding is an emergency medical condition. Herein, we present our use of this two-stage InterNet model for detection of active abdominal arterial bleeding using emergency DSA imaging.

**Methods:**

Firstly, 450 patients who underwent abdominal DSA procedures were randomly selected for development of the region localization stage (RLS). Secondly, 160 consecutive patients with active abdominal arterial bleeding were included for development of the bleeding site detection stage (BSDS) and InterNet (cascade network of RLS and BSDS). Another 50 patients that ruled out active abdominal arterial bleeding were used as negative samples to evaluate InterNet performance. We evaluated the mode's efficacy using the precision-recall (PR) curve. The classification performance of a doctor with and without InterNet was evaluated using a receiver operating characteristic (ROC) curve analysis.

**Results:**

The AP, precision, and recall of the RLS were 0.99, 0.95, and 0.99 in the validation dataset, respectively. Our InterNet reached a recall of 0.7, the precision for detection of bleeding sites was 53% in the evaluation set. The AUCs of doctors with and without InterNet were 0.803 and 0.759, respectively. In addition, the doctor with InterNet assistant could significantly reduce the elapsed time for the interpretation of each DSA sequence from 84.88 to 43.78 s.

**Conclusion:**

Our InterNet system could assist interventional radiologists in identifying bleeding foci quickly and may improve the workflow of the DSA operation to a more real-time procedure.

## Introduction

Active abdominal arterial bleeding is a medical emergency that may lead to haemorrhagic shock or circulatory instability if left untreated (1–5). Clinicians experience difficulty in dealing with this complicated condition (2, 5, 6). Most cases of active abdominal arterial bleeding are medically treated by correcting coagulation abnormalities or through endoscopy (7–9). Nonetheless, these methods can fail in some patients with significant bleeding, in which cases endovascular treatment is desired (3, 10–14). Due to its advantages of reduced morbidity and mortality, endovascular treatment using digital subtraction angiography (DSA) is now preferred over open surgery (5, 11, 15–17).

Rapid and accurate diagnosis of arterial bleeding by an interventional physician *via* DSA remains challenging (1). Human limitations in a crowded tertiary hospital include staff shortage, excess workload, and, especially, a lack of knowledge among radiologists regarding arterial bleeding. Under these circumstances, an automated system is needed to alleviate the tedious task of screening out incidental findings and allowing physicians more time to interact with patients and other health care providers. Further, such a system would help address the lack of expert radiologists in rural and community hospitals. What's more, the bleeding could be subtle in some cases. It is difficult to identify subtle bleeding by human quickly, and it is more difficult for junior doctor. So, one of the important values of our system is to shorten diagnosis time and to reduce the rate of missed bleeding sites. Deep learning approaches have provided exciting solutions medical image in medical image detection. The diagnosis of bleeding involves a typical computer visual task of classification of radiological images into bleeding and non-bleeding categories and detection of bleeding sites. However, computer-assisted automated detection of active abdominal arterial bleeding from DSA images has not been previously reported.

In current practice, a captured video sequence is reviewed offline by the physician to identify bleeding sites before the intervention is performed. A usable AI (artificial intelligence) system should be able to replace this offline review with automated detection of bleeding sites. Thus, our system was designed and evaluated based on this first goal. However, the current workflow must ultimately be improved to a more real-time system ideally. If the automated system detects bleeding sites correctly in most frames and at the video frame rate, there might be no need for an offline review. The physician could directly view the highlighted bleeding sites in real-time and perform the surgery, which would reduce the surgery time. In this work, we proposed a two-stage deep learning model (named *InterNet*) for real-time detection of active abdominal arterial bleeding using emergency DSA imaging. We hypothesized that the InterNet can detect active abdominal arterial bleeding at a faster speed and higher sensitivity.

## Materials and Methods

### Data Acquisition

Firstly, 450 patients who underwent abdominal DSA procedures were randomly selected from our PACS system for development of the region localization stage (RLS). Secondly, 160 consecutive patients with active abdominal arterial bleeding who underwent endovascular treatment between January 2013 and January 2020 were retrospectively included for development of the bleeding site detection stage (BSDS) and InterNet (cascade network of RLS and BSDS). These 160 patients had clinical signs of active abdominal arterial bleeding: blood from a postoperative drainage tube, haematuria, haematochezia, hypotension, tachycardia, or a low hemoglobin level. Another 50 patients who underwent abdominal DSA procedures that ruled out active abdominal arterial bleeding were randomly selected and used as negative samples to evaluate InterNet performance.

A standard transfemoral approach was used in all angiographic procedures. A sheath introducer was placed in the right or left common femoral artery using the Seldinger technique. Selective angiography of the abdominal aortic branches was performed using a 5-Fr catheter in all patients. Super selective angiography of the tiny branches was performed using a microcatheter.

DSA images usually contain multiple sequences, and each sequence consisted of 30–50 video frames at six frames per second. All data were stored in Digital Imaging and Communications in Medicine (DICOM) format. All data were manually annotated using LabelImge software (GitHub, Inc., San Francisco, CA, USA). The bleeding sites and angiographic regions were manually segmented and annotated by two radiologists. The segmented images were then reviewed by another experienced radiologist. Any disagreements in segmentation were resolved through consensus among the three radiologists.

### Dataset Splitting

A total of 546 sequences from 450 patients were used for RLS development. These patients were randomly split into a training dataset (80%) and a validation dataset (20%). From the 160 patients with active abdominal arterial bleeding, 182 sequences from 90 patients were classified into the BLDS training dataset; 49 sequences from 20 patients were classified as a validation dataset for stability and generalizability of the RLS and BSDS cascade network (InterNet). Sixty-seven sequences from 50 actively bleeding patients and 80 sequences from 50 patients without active bleeding were classified as an independent testing dataset for InterNet.

### Deep Learning Model Development

**Figure 1:**
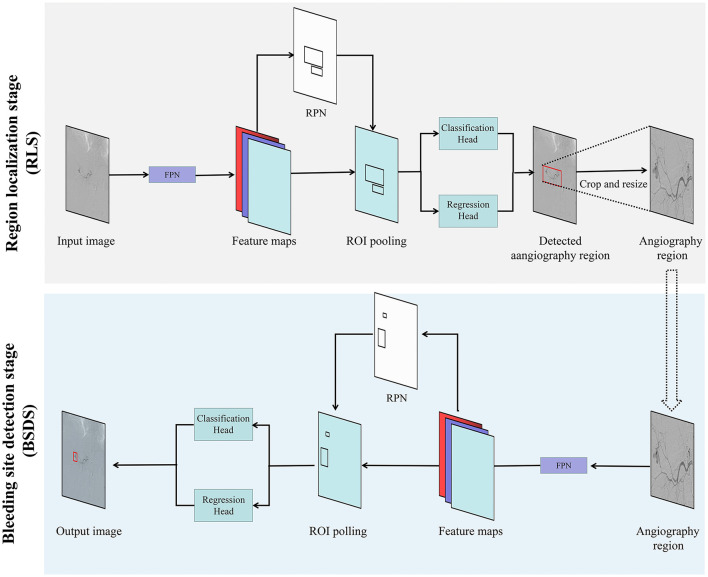
Overview diagram of the proposed two-stage deep learning approach (InterNet). The system first detected the angiographic region from the original frame image. The output of the RLS was used as input in the next stage of redundancy reduction. RPN, region proposal network; ROI, region of interest; FC, fully connected layer; Bbox, bounding box.

The entire program was performed with Pytorch version 1.2 (Pytorch, Warsaw, Mazowieckie, Poland) as the backend, on a desktop computer equipped with an Intel (R) Xeon(R) Silver 4110 system (Intel Inc., Santa Clara, CA, USA), 64 GB RAM, and a GeForce RTX 2080Ti GPU (Nvidia, Santa Clara, CA, USA). The InterNet detection system was developed to automatically detect bleeding sites on DSA images using a two-stage process, first localizing the angiographic region from the original frame image (RLS), followed by bleeding site detection on the cropped image (BSDS). The framework of our two-stage detection system is schematized in Figure 1. The RLS was based on the sparseness of bleeding sites in a sequence and within a frame image. ResNet50 was used as the backbone for our two-stage deep learning model framework.

**Figure 2:**
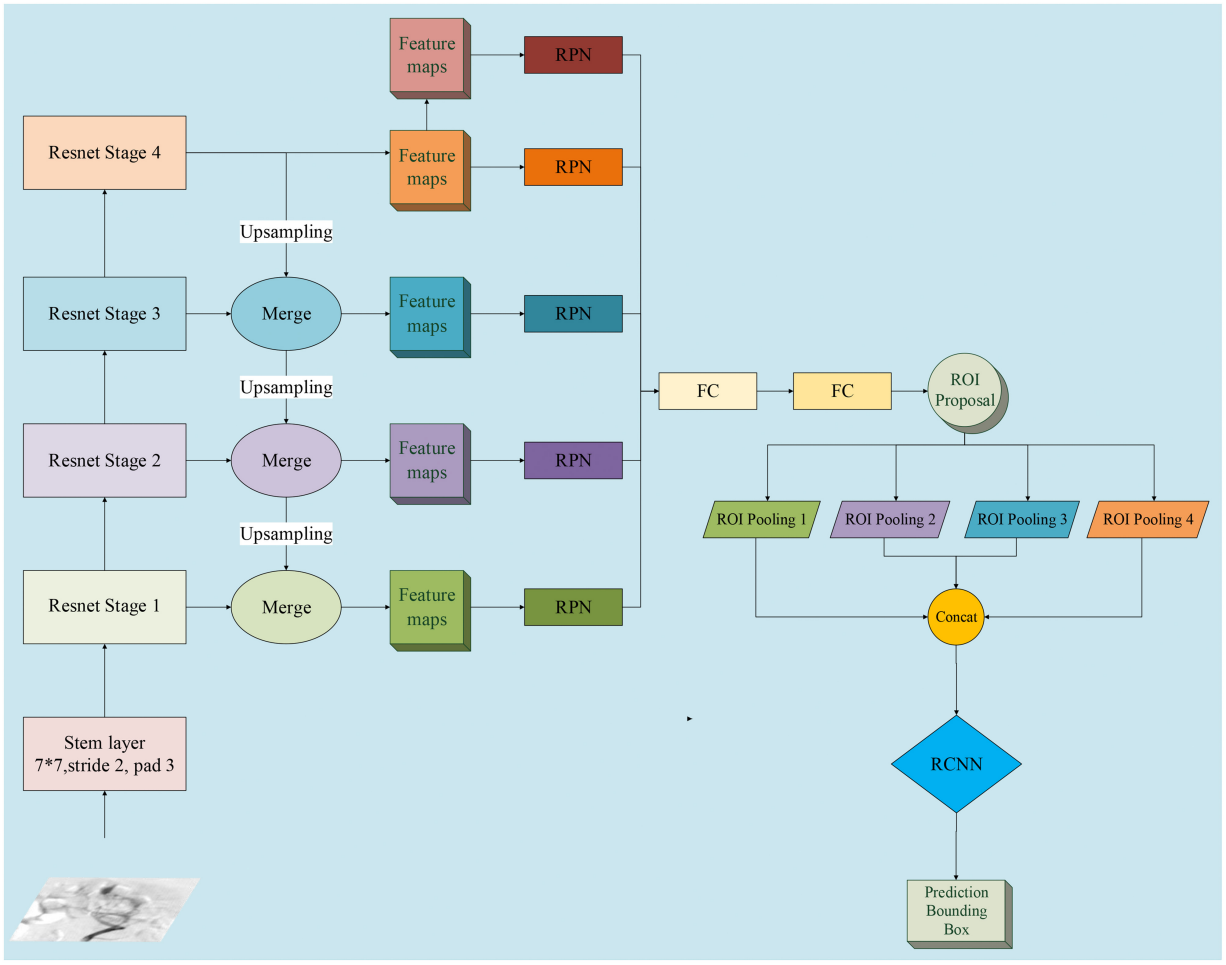
Detailed network structure diagram of feature pyramid network (FPN). We adopted FPN with a ResNet-50 backbone for InterNet. FPN is an outstanding detector for many visual tasks and can identify objects at multi-scales. RPN, region proposal network; ROI, region of interest; FC, fully connected layer; bbox, bounding box; RCNN, Region Based Convolutional Neural Networks.

Multi-scale features were extracted to create feature maps, and the region proposal networks (RPNs) were applied to generate region proposals *via* classification and regression (18). The proposed regions underwent non-maximum suppression to filter the highly overlapping regions. Region pooling unified the various-sized regions to the same size. The resulting region candidates were put through the Region Based Convolutional Neural Networks (R-CNN). The targets were classified, and the bounding boxes underwent a second regression to achieve the final target detection. Moreover, we applied the feature pyramid networks (FPN) on the framework of our two-stage detection system (19). The cost and benefit of using the FPN compared to the approach without FPN was also evaluated. The detailed network structure of multi-scale features extraction is schematized in Figure 2.

To tune the detection system, we adjusted the size of the input image. According to the detection performance, we chose the optimal value of the key parameter of “resize.” To avoid overfitting, we used common techniques to augment the data. Contrast-limited adaptive histogram equalization (CLAHE) was applied to reduce the intensity range, followed by random shift and rotation to augment the orientation and position of the bleeding site samples (20). Perturbation of intensities and contrast and a random median filter were applied to improve the distribution of the samples.

### Performance Assessment Between Doctors With and Without InterNet Assistant

To evaluate the benefit of InterNet, we compared the classification efficiency between doctors in terms of patients with InterNet assistant using the independent testing dataset. The classification performance and elapsed time were recorded.

### Statistical Analyzes

We evaluated the model's efficacy using the precision-recall (PR) curve, which is commonly used to show the compromise between precision and recall. By moving along the curve, various compromises between precision and recall can be acquired, enabling us to choose between the two. A high recall indicates a higher rate of detection (fewer false negatives), and a high precision indicates a lower rate of false positives. The average precision (AP) was used to evaluate the detection precision of the deep learning algorithms. A prediction is considered to be true positive if Intersection over Union (IoU) > 0.5, and false positive if IoU < 0.5. The frame-per-second (FPS) rate of each test was calculated to evaluate whether the bleeding sites could be tracked in real-time. The classification performance of a doctor with and without InterNet was evaluated using a receiver operating characteristic (ROC) curve analysis. The area under the curve (AUC), sensitivity, and specificity were calculated. The differences in elapsed time for a doctor with and without InterNet were compared using the Mann-Whitney *U*-test. Statistical analyses were performed using R (version 3.3.4, http://www.Rproject.org). The threshold for statistical significance was set at a two-sided *p* < 0.05.

## Results

We used the area calculated from the segmented mask of each positive DSA image to represents the amount of bleeding. The mean bleeding site area of the 67 sequences from 50 actively bleeding patients in the independent testing dataset is 938.4 ± 1,707.1 square millimeter. Among the 50 patients, the bleeding locations of 24 cases are in kidney; 19 cases are in digestive tract; three cases are in spleen; two cases are in uterus; and two cases are in other organs.

The AP, precision, and recall of the RLS were 0.99, 0.95, and 0.99, respectively. This means that the angiographic region could be correctly recognized in 99 out of 100 testing images. The P-R curve of the RLS on the validation dataset is shown in Supplementary Figure 1.

**Figure 3:**
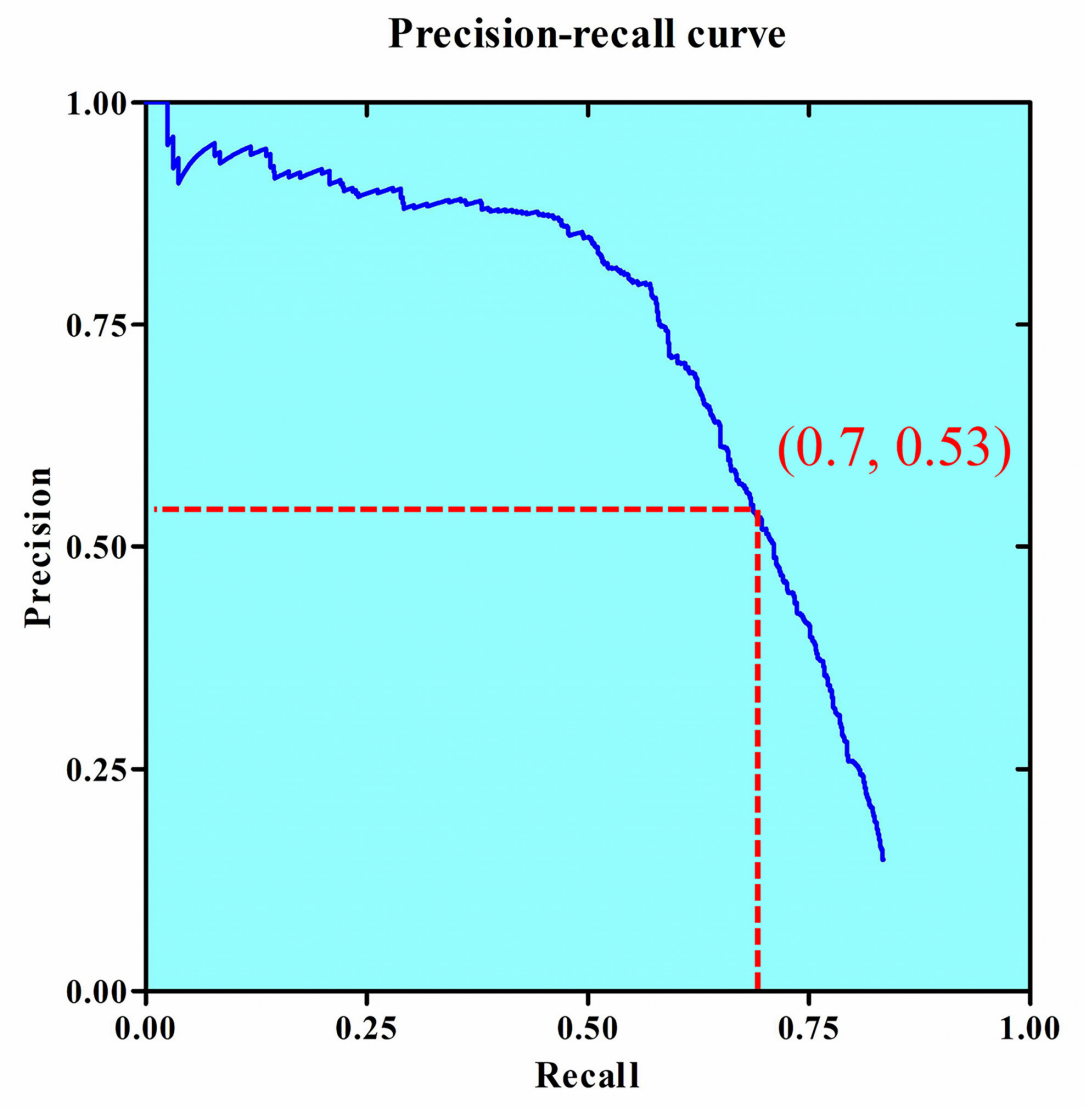
The precision–recall curve of the InterNet. The average precision of the InterNet was 64.5 on the evaluation dataset. We selected a spot with a recall of 0.7 and a precision of 0.53.

The baseline system showed an AP of 58.1% and FPS rate of 4.2, while the network with FPN showed improved AP of 60.3% and FPS rate of 5.0. The detection results for the system on the validation dataset with and without FPN are shown in Table 1. The key parameter of “resize” was found to be optimal at 1,333 × 800. The AP reached 64.5% with this input image size, while the FRS showed a slight decrease from 5.0 to 3.9. The model including Baseline + FPN and resize 1,333 × 800 was selected as the final structure for our InterNet system. The effect of changing the resize parameters of the detection system is shown in Table 2. The InterNet P-R curve for the evaluation dataset is shown in Figure 3. For the task of detection, a high recall was more desirable than a high precision. Therefore, we picked a spot with a recall of 0.7 and precision of 0.53.

**Table 1:** Effect of feature pyramid networks of the detection system.

	**Baseline**	**Baseline + FPN**
AP (%)	58.1	60.3
FPS	4.2	5.0

*AP, Average Precision; FPS, frames per second*.

**Table 2:** Effects of changing resize parameters of the detection system.

	**Baseline + FPN**	**Baseline + FPN**
	**(Resize 1,024 ×512)**	**(Resize 1,333 ×800)**
AP (%)	60.3	64.5
Precision	0.514	0.531
Recall	0.683	0.703
FPS	5.0	3.9

*FPN, Feature Pyramid Network; AP, Average Precision; FPS, frames per second*.

**Figure 4:**
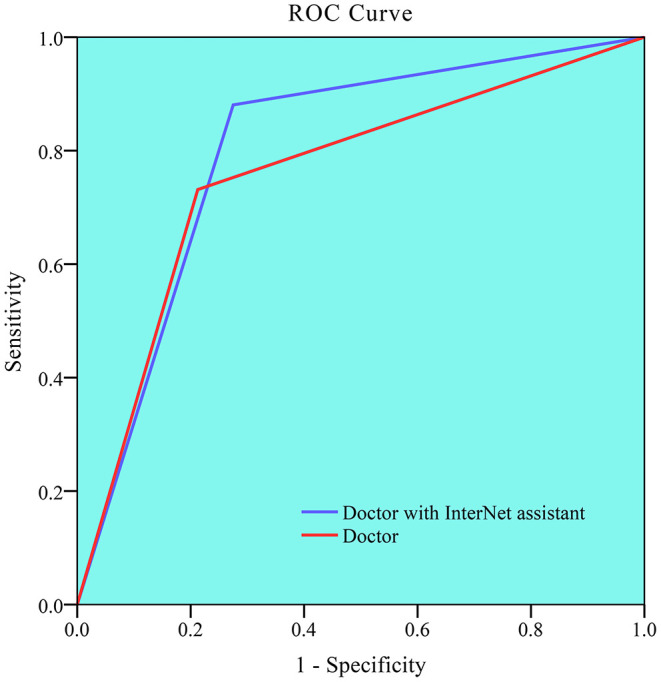
Receiver operating characteristic (ROC) curve for doctor without and doctor with InterNet assistant. Doctor with InterNet assistant showed a superior performance to that of doctor without InterNet assistant. The AUCs of doctor with and doctor without InterNet were 0.803 and 0.759, respectively.

**Figure 5:**
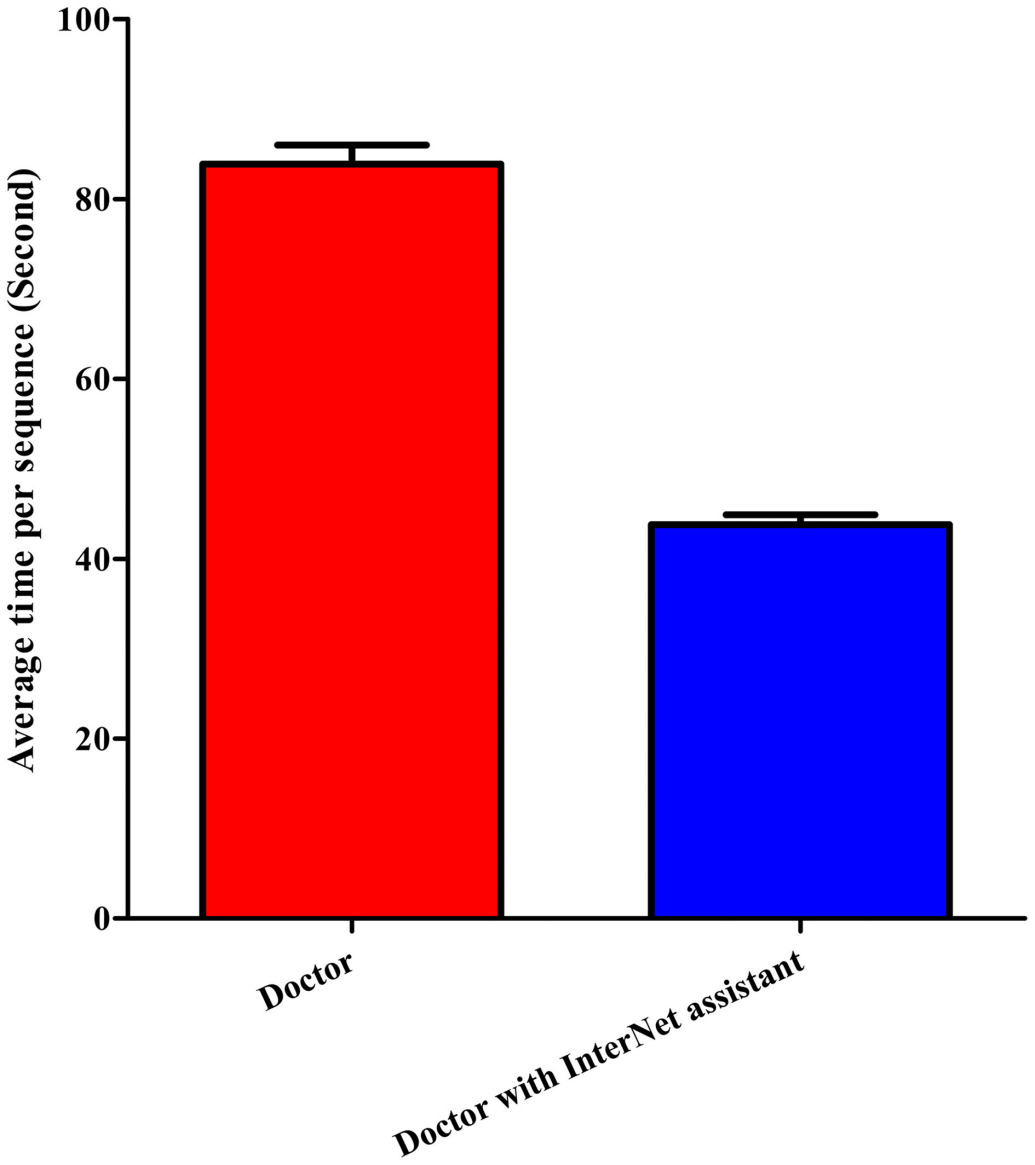
Elapsed time of doctor without and doctor with InterNet assistant. Doctor with InterNet assistant significantly reduced the elapsed time for the interpretation of each DSA sequence from 84.88 to 43.78 s per sequence.

**Figure 6:**
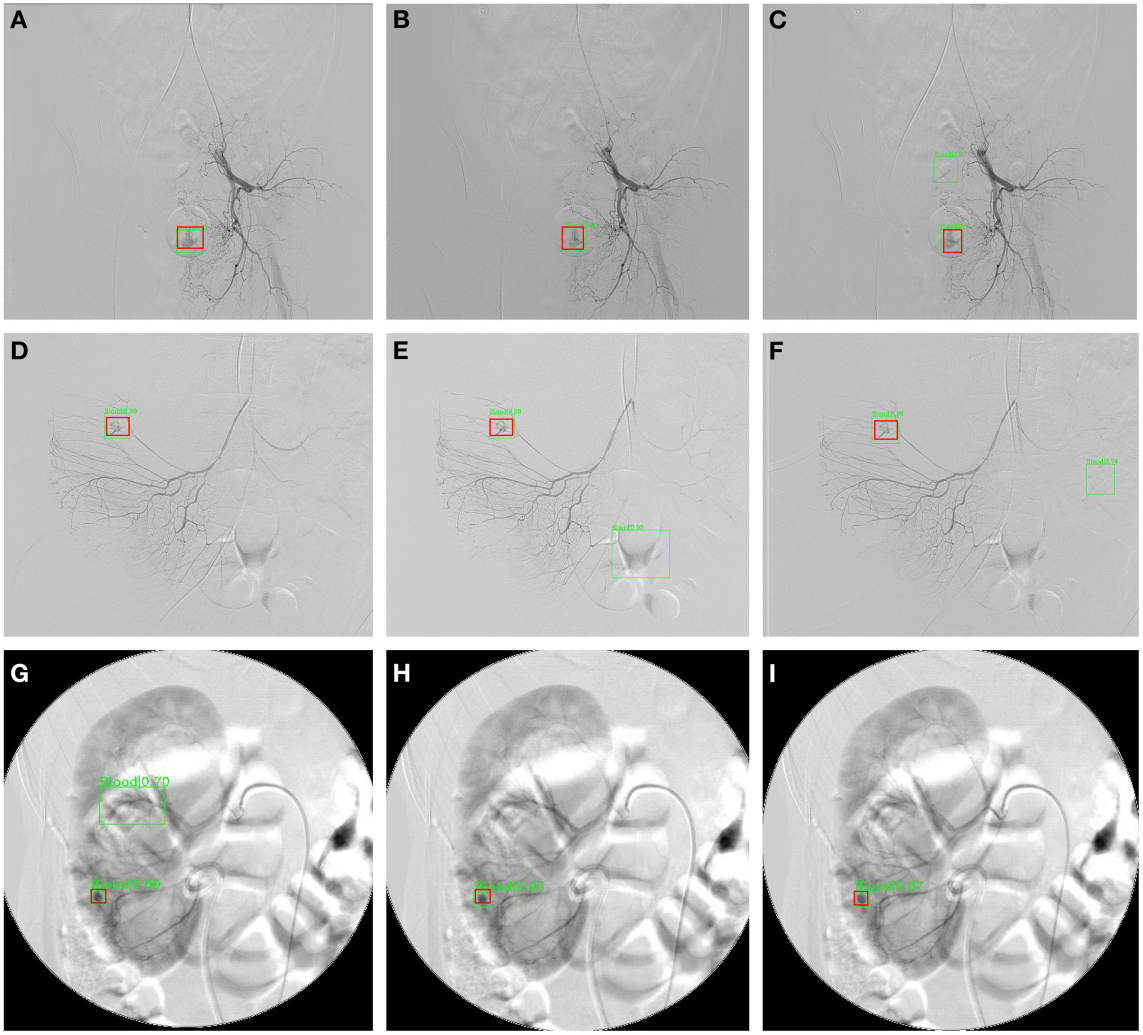
Three sample results from InterNet. **(A–C)** Are images from a patient with subrectal arterial branch bleeding; **(D–F)** are images from a patient with bleeding from a right colonic artery branch; **(G–I)** are images from a patient with a bleeding branch of the inferior artery of the right kidney. The red boxes represent the ground truth bleeding bounding boxes; the green box represents the detected bleeding bounding box.

Table 3 and Figure 4 summarize the classification performance of a doctor with and without InterNet. The doctor with InterNet showed a superior performance to that of the doctor without InterNet. The AUCs of doctors with and without InterNet were 0.803 and 0.759, respectively. In particular, the doctor with InterNet assistant showed a substantially increased sensitivity, from 73.17 to 88.06%. In addition, the doctor with InterNet assistant could significantly reduce the elapsed time for the interpretation of each DSA sequence from 84.88 to 43.78 s per sequence (*p* < 0.01; Figure 5). Examples of the prediction results obtained by our proposed InterNet are shown in Figure 6.

**Table 3:** Classification performance of a doctor with and without InterNet.

	**Doctor with**	**Doctor without**
	**InterNet assistant**	**InterNet assistant**
AUC	0.803	0.759
Sensitivity (%)	88.06	73.13
Specificity (%)	72.50	78.75
Accuracy (%)	80	76
PPV (%)	73.00	74.00
NPV (%)	88.00	78.00
Time (second/sequence)	43.78	84.88

*AUC, area under the receiver operating characteristic curve; PPV, positive predict value; NPV, negative predict value*.

## Discussion

Given the efficacy and safety of transcatheter arterial embolization compared with open surgery for the treatment of active abdominal arterial bleeding (5, 11, 15–17), accurate and rapid detection of bleeding sites is the key to success of transcatheter arterial embolization. In this study, we built an automated system based on a deep neural network model to detect active abdominal arterial bleeding on DSA images. Our InterNet system could help doctors in making a faster and more accurate interpretation. To our knowledge, this is the first system to automatically detect active arterial bleeding sites in DSA images.

In this study, we adopted two-stage deep learning for detection of active abdominal bleeding sites. In RLS, the angiographic region is proposed for detecting potential bleeding sites. In this stage, our detection system located a specific region to reduce the interference from other regions. The output of the RLS was used as input in the next stage of redundancy reduction. A practical benefit of RLS is that any data sequence, whether positive or negative, can be used for training the network for angiographic region extraction. This along with the ease of ROI labeling creates ample data to train a robust algorithm to extract the angiographic regions from the original frame images.

In the current study, we adopted FPN with a ResNet-50 backbone for the InterNet, because FPN is an outstanding detector for many visual tasks. FPN is capable of multi-scale feature extraction, which fits well with the task of detecting bleeding sites that have large variations in their sizes and shapes. FPN has a top-to-bottom pathway in addition to the bottom-to-top pathway of a regular neural network; hence, the semantic information from the top levels helps enhance the detailed information in the lower layers, leading to a powerful multi-scale capacity (19). In our study, the baseline system with FPN showed a relatively higher compared without FPN (60.3 vs. 58.1%).

Embolization requires the localization of bleeding sites, which can be easily missed by a physician. For our abdominal bleeding detection task, a low false negative rate is more desirable than a low false positive rate, since for a physician, it is easy to miss both, a bleeding spot and to rule out one. For this task of detection, a high recall was more desirable than a high precision. Therefore, we picked a spot with a recall of 0.7. At a recall of 0.7, the precision for detection of bleeding sites was 53% in the evaluation set. A recall of 70% means that the bleeding spot will be revealed to the physician in two of the three frames. A precision of 53% means that, on an average, for every correctly detected bleeding site, there will be <1 false detection. This false positive rate should be acceptable and not divert much of the physician's attention. The doctor with InterNet performed superiorly to the doctor without InterNet. In addition, the doctor with InterNet assistant could significantly reduce the elapsed time for the interpretation of each DSA sequence.

In present-day DSA surgery, after the DSA sequences are acquired, the physician reviews the sequence offline to detect the bleeding sites before performing the intervention. This workflow does not require our system to have real-time performance to replace the physician's effort of detecting bleeding sites. A more advanced use of the system would be to improve the workflow of the DSA operation to a more real-time procedure, thus eliminating the need for offline review and discussion. Ideally, the physician would look at the overhead monitor and observe the DSA images with overlaid marks of automatically detected bleeding sites (Supplementary Figure 2). To achieve this, it is best for the system to reach six frames per second—the frame rate of the captured imaging sequence. The frame rate achieved with Python in this study is close to 4 frames per second, and it is conceivable that a product based on optimized C++ code should reach six frames per second without much difficulty. In such a system, the physician could watch the video sequence in real-time. The system will produce some false positives, with an average of one false positive every two frames due to a precision of 53%. The bleeding sites in ~1 of 3 frames will not be marked due to a recall rate of 70%. Despite these imperfections, at an FRS of 4, the physician should be able to mentally make up the gap frames and eliminate the false marks with ease.

In recent years, many deep learning approaches have been developed for medical imaging analysis (21–23). A few studies have applied deep learning in DSA imaging. Alexander et al. trained a CNN system to automatically segment saccular aneurysms (pre- or post-coiling) and surrounding vasculature from DSA images (24). Yufen used residual density to generate a DSA image from a single live image without mask data acquisition, thus avoiding the appearance of motion artifacts in the image (25). To date, no study has applied the deep learning system for the detection of bleeding in DSA. We suspect that the difficulty in obtaining sufficient data is an important factor limiting its application to DSA. In this study, we applied deep learning for the detection of bleeding on DSA for the first time. Further applications of deep learning in DSA should be proposed and evaluated in future work. Deep learning may play an important role in surgery.

The main clinical applications of the proposed method are as followings. First, with the help of the current system, the physician would reduce the rate of missed bleeding sites, especially when the bleeding is subtle. Missed bleeding sites could lead to poor outcomes, and some patients may need a second procedure. Therefore, our system has the potential to improve the prognosis of patients. Second, the deep learning system developed in this study has the potential to shorten operation time, which may also reduce the radiation dosage to doctors and patients during the operation (26). Third, the automated system in our study would help address the lack of expert radiologists in rural and community hospitals. The CT Angiography (CTA) is also a common method for diagnosing active bleeding abdomen bleeding. Compared to CTA, DSA play import roles not only in diagnosis but also in appropriate management of abdomen bleeding. Most of the cases included in current study were performed emergency DSA surgery, therefor very few patients underwent CTA before DSA due to limited time. For the 50 actively bleeding patients in the independent testing dataset for InterNet, only seven patients underwent CTA before DSA surgery. The two radiologists did not view their CTA results when identify the bleeding sites. Thus, the CTA examination would not influence the research results in this retrospective study.

This study has several limitations. Firstly, this was a retrospective study design at a single institute. The number of images with bleeding sites included in the test set was also not very large. Secondly, only DSA imaging of one manufacture was included. Based on current results, we could not sure whether the method could be generalized to various DSA sequences from various manufactures. Therefore, our system should be validated in multicentre studies of a larger scale. Thirdly, only abdominal bleeding was included. Other types of bleeding (neck or thoracic bleeding) were not included because of their low incidences.

In this study, we presented a two-stage model InterNet for active abdominal bleeding detection using deep learning with DSA data. This work has created a usable system to automatically detect bleeding sites in DSA sequences. Our developed InterNet system could help doctors in achieving a faster and more accurate interpretation. A prospective clinical trial is necessary to determine the effectiveness of this system and whether it will ultimately improve patient care and outcomes.

## Data Availability Statement

The raw data supporting the conclusions of this article will be made available by the authors, without undue reservation.

## Ethics Statement

The studies involving human participants were reviewed and approved by Institutional Review Board of Tongji Hospital of Huazhong University of Science and Technology. Written informed consent for participation was not required for this study in accordance with the national legislation and the institutional requirements.

## Author Contributions

XM, ZF, and NW: conception and design. PZ and XM: collection and assembly of data. SC, TF, HS, and JG: data analysis and interpretation. XM, ZF, JG, and NW: manuscript writing. All authors: final approval of manuscript.

## Funding

This paper was supported by the National Natural Science Foundation of China under Grant No. 81801668 and 61773408.

## Conflict of Interest

SC, TF, and HS were employed by United Imaging Intelligence. The remaining authors declare that the research was conducted in the absence of any commercial or financial relationships that could be construed as a potential conflict of interest.

## Publisher's Note

All claims expressed in this article are solely those of the authors and do not necessarily represent those of their affiliated organizations, or those of the publisher, the editors and the reviewers. Any product that may be evaluated in this article, or claim that may be made by its manufacturer, is not guaranteed or endorsed by the publisher.
