# Supplementary material for: InterNet: Detection of Active Abdominal Arterial Bleeding Using Emergency Digital Subtraction Angiography Imaging With Two-Stage Deep Learning
Source: Front Med (Lausanne). 2022 Jun 29;9:762091. doi: 10.3389/fmed.2022.762091 (PMC9276930; doi:10.3389/fmed.2022.762091)
Supplement: Supplementary file 1 [file Table_1.DOCX]

Supplementary Material

# Supplementary Figures

**
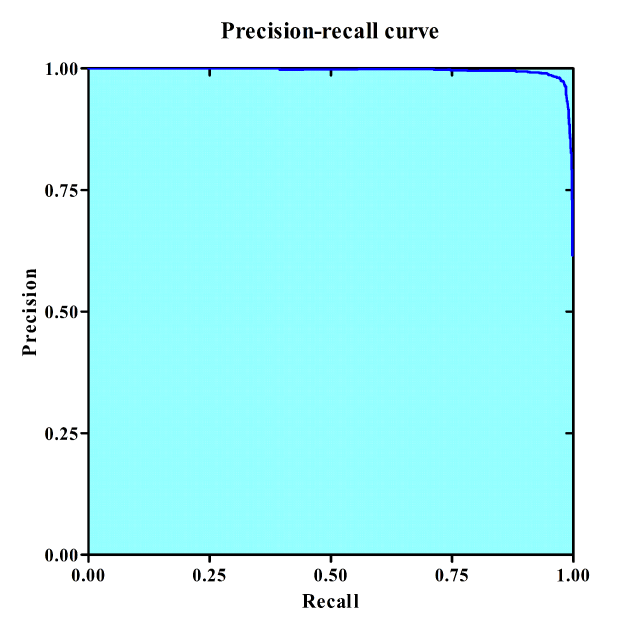
**

**Supplemental Figure. 1. Precision–recall curve of the region localisation stage (RLS).** The average precision of the RLS was 0·99 in the evaluation dataset. We selected a spot with a recall of 0·95 and a precision of 0·99.


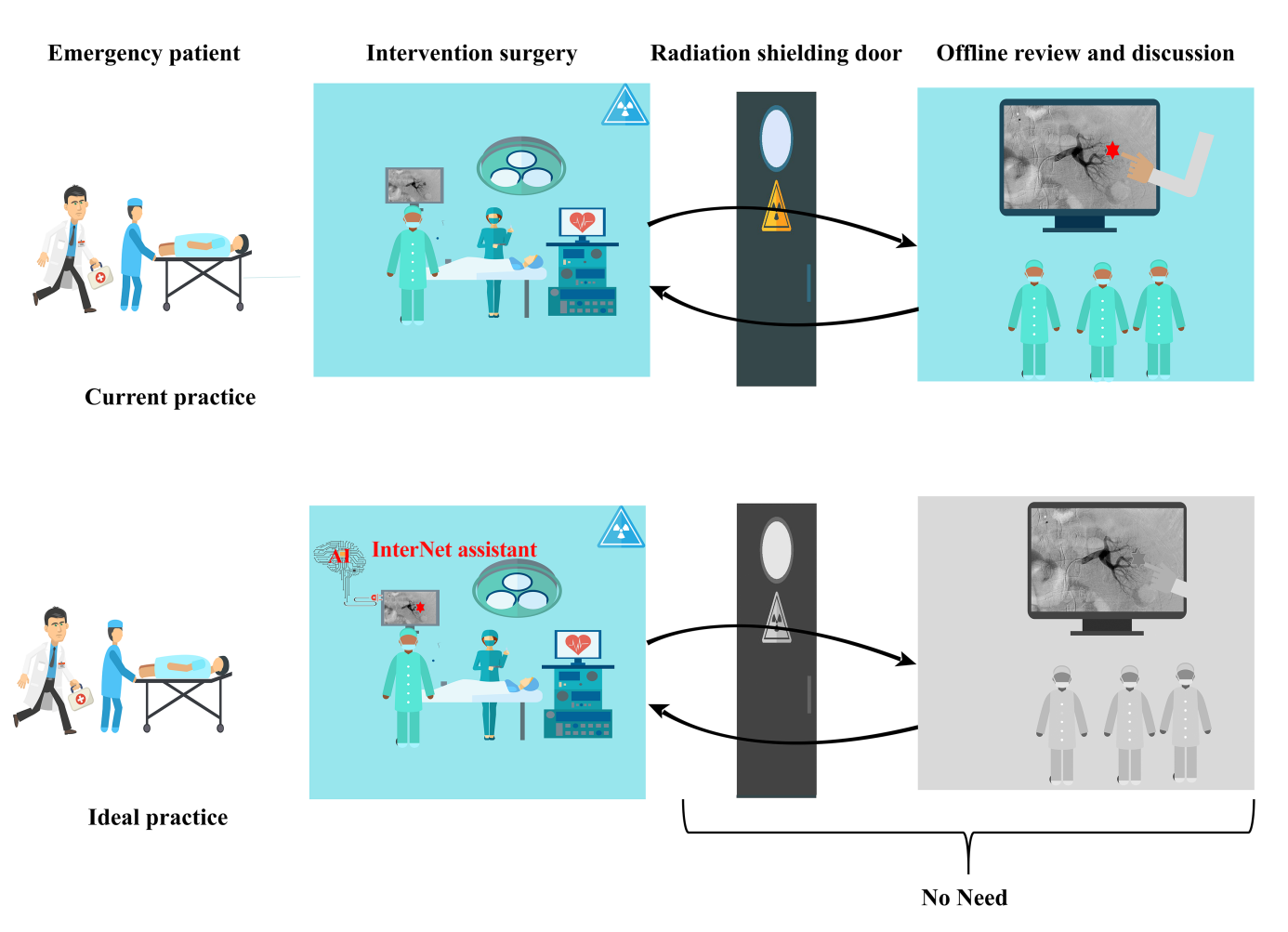


**Supplemental Figure. 2. Current and ideal practices of DSA operation.** At present, after the DSA sequences are acquired, the physician reviews the sequences offline to detect bleeding sites before performing the intervention. Ideally, the physician would observe the DSA images on an overhead monitor with marks overlaying the automatically detected bleeding sites, thus eliminating the need for offline review and discussion.
